# Social contexts of fertility desire among non-childbearing young men and women aged 15–24 years in Nigeria

**DOI:** 10.1186/s12978-021-01237-1

**Published:** 2021-09-20

**Authors:** Joshua O. Akinyemi, Clifford O. Odimegwu

**Affiliations:** 1 Department of Epidemiology and Medical Statistics, Faculty of Public Health, College of Medicine, University of Ibadan, Ibadan, Nigeria; 2 Demography and Population Studies Programme, Schools of Public Health and Social Sciences, University of the Witwatersrand, Johannesburg, South Africa

**Keywords:** Fertility desire, Desired family size, Ideal number of children, Social contexts, Individual characteristics, Contextual factors, Demographic and Health Surveys, Nigeria

## Abstract

**Background:**

Reduction in ideal number of children has been suggested as a necessary precursor for fertility decline especially in high fertility countries of Western and Central Africa. In this study, we explored the social contexts of fertility desires by documenting the effects of individual, household as well as contextual characteristics among young men and women in Nigeria.

**Methods:**

Data source was the male and female recode file of 2018 Nigeria Demographic and Health Survey. Analytical sample comprised 2674 males and 9637 females aged 15–24 years. The main outcome variable was desire for large family size (DLFS) defined as ideal number of children greater than four. Analysis involved use of descriptive statistics and random-effect logit models fitted in four stages.

**Results:**

DLFS was 71% among young men and 53% in women. Individual-level factors associated with DLFS among men includes Islam religion (OR = 3.95, CI 2.68–5.83), household size (OR = 1.05) and richer (OR = 0.47, CI 0.29–0.75) or richest wealth index (OR = 0.28, CI 0.16–0.75). Geo-political region and high level of negative attitude to family planning (OR = 1.72, CI 1.23–2.40) were the main contextual factors associated with DLFS. For women, individual-level correlates were education, religion, ethnicity, marital status, household size, and wealth index. Contextual factors include geo-political region, community education (OR = 0.68, CI 0.52–0.89), child mortality experience (OR = 1.29, CI 1.11–1.51) and negative attitude to family planning (OR = 1.36, CI 1.13–1.65). The influence of religion, household wealth and attitude to family planning differ between young men and women.

**Conclusion:**

Active communication and programmatic interventions are needed so that desire for large family size by young men and women do not become a clog for fertility transition in Nigeria.

## Plain Language Summary

Reduction in ideal number of children has been suggested as a necessary condition for fertility decline especially in high fertility countries of Western and Central Africa. In this study, we explored the effects of individual, household as well as community characteristics on fertility desires among young men and women aged 15–24 years in Nigeria. We analysed data for 2674 males and 9637 females aged 15–24 years extracted from the 2018 Nigeria Demographic and Health Survey. The outcome variable was desire for large family size (DLFS) defined as ideal number of children greater than four. Results showed that DLFS was 71% among men and 53% in women. Individual-level factors associated with DLFS among men include Islam religion, household size and wealth status. Geo-political region and high level of negative attitude to family planning were the main community-level factors associated with DLFS. For women, individual-level positively associated with DLFS were Islam religion, and being currently married. Compared to Yoruba, other ethnic groups were more likely to favour DLFS. The negative factors associated with DLFS among young women include higher education and wealth status. At the community-level, Northern geo-political regions, child mortality experience and negative attitude to family planning were positively associated with DLFS. The influence of religion, household wealth and attitude to family planning differ between young men and women. Multi-dimensional strategies with active communication and programmatic interventions are needed so that desire for large family size by young men and women do not slow down fertility transition in Nigeria.

## Background

Demographic evidence suggests a positive correlation between fertility levels and desired family size [DFS] [1, 2]. By implication, fertility decline can be realized if there is reduction in DFS. While total fertility rate in many countries of Eastern and Southern Africa is following a downward trend, those of Western and Central Africa has stagnated at about 6.0 as of 2015 [3]. This is not to say that the sub-region never experienced transition, but the speed is much slower than expected due to two major factors. The first is low uptake of family planning with minimal improvement over time [4]. The second factor which even impact negatively on family planning is fertility desire which is predominantly pronatal—strong cultural norm and belief in having a large number of children [5].

The demographic and socio-economic correlates of DFS include age, parity, child mortality, Islamic religion, educational attainment and exposure to mass media [6, 7]. At the community level, poverty, low education and high level of child mortality have been implicated [8]. Specifically, results from ecological analysis of determinants of desired family size in sub-Saharan Africa revealed that it is negatively correlated with percentage of women with secondary education as well as childhood mortality rate [6, 7]. This evidence is in tandem with fertility transition theory which argues that fertility decline is associated with socio-economic development [9, 10]. Urbanization, education, access to information, employment opportunities are all indices of socio-economic development and they can also be referred to as characteristics of the social context.

The socio-economic contexts, family and childbearing norms are particularly important. For example, the role of men as family heads cannot be discountenanced in fertility decision by women [9]. Some previous studies have documented the importance of social contexts for reproductive health behaviours such as multiple sex partnership, contraceptive use among adolescents and youth in Sub-Saharan Africa (SSA) [8, 11–13].

Majority of the previous studies that explored the correlates of fertility desires have been conducted among broad reproductive age group 15–49 years [2, 14, 15]. Could the desires of young men and women be different especially considering that they have not been exposed to the burdens associated with childbearing? This is a very important question for population programmes because evidence shows that transition to low fertility is contingent on downward review of desired family size [16, 17]. Given the prevalent unfriendly developmental environment in which young people in SSA have found themselves, are they also biased in favour of large families?

Evidence on fertility desire is important for the design and implementation of family planning programmes especially for young persons who are very strategic to the prospect of transition in high fertility settings of SSA. Fertility desires of young people can affect their reproductive health behaviour through the life course [7]. During this period of transition from dependence on parents to independent adult life and eventual family formation, reproductive health behaviour may be easier to influence. In addition, fertility among young persons has inter-relationship with sustainable development goals such as gender equality, women empowerment, eradication of hunger and poverty [18]. For instance, uncontrolled fertility and population growth can negatively affect the quest to eradicate hunger and poverty. In contrast, gender inequality and women empowerment have a direct influence on fertility levels [19].

Nigeria is a suitable setting to interrogate this issue because of its peculiar fertility, socio-cultural and economic characteristics. It is the most populous country in Central and Western Africa with TFR changing slightly from 6.3 in 1990 to 5.3 in 2018 [20]. Fertility profile is heterogeneous across the six geo-political regions but there is some level of homogeneity among states within the regions [21]. Data from 2018 Nigeria Demographic and Health Survey (NDHS) showed that the average DFS among women (married and unmarried) across the six regions were: North West-7.5, North East-7.5 North Central-5.7, South East-5.0, South South-4.6, South West-4.0 [20]. There is a similarity in the distribution of ethnicity and geo-political regions such that Hausa and Fulani are the dominant groups in North East and North West. In the South East are mostly Igbos while South West is mostly Yorubas. All the ethnic groups are vastly pronatal but it’s stronger in the Northern regions especially among Hausas and Fulanis. Nigeria has a population policy first published in 1988 and later revised in 2004. One of the key fertility targets in these policies was to ensure that by year 2015, women have at most four children in their reproductive life [22]. Assessment of the 2004 policy showed that many of the targets were not achieved [23, 24]. Another review of the policy is long overdue.

Few previous studies have explored factors associated with fertility desires in Nigeria but were conducted among either married men and women of reproductive age [25–27] or among couples [28, 29]. For instance, fertility decision making in Nigeria is dominated by men and their desire for large number of children is influenced by ethnicity, religion, education and spousal communication [25, 29]. With respect to ethnicity, Hausa/Fulani men desired larger family sizes than Igbo, Yoruba and other ethnic groups. The desired family size was smaller among Christians compared to other religions. Further, evidence from a qualitative study among married women also showed that ideal number of children was persistently high and affected by religion and cultural beliefs such as propagation of family lineage; children as old-age security and communal prestige/respect [30]. A qualitative study on childbearing motivations among young people aged 15–19 irrespective of marital status in North West Nigeria shows that adolescents also believed in large families and their motivations for such are often due to parental pressure and social norms in their communities [31].

It is in view of these dynamics that the present study is aimed at exploring the relationship between social contexts and fertility desires among young adults in Nigeria. The individual, household and contextual characteristics associated with fertility desires are documented and the implications for fertility transition are highlighted.

## Methods

### Data source

This study was based on data extracted from the 2018 Nigeria Demographic and Health Survey (NDHS). In the NDHS, national representative samples of households were selected via two-stage stratified cluster sampling technique [20]. Subsequently, women aged 15–49 years (n = 41,821) in selected households were interviewed. Men aged 15–59 years (n = 13,311) were selected from one-third of households. Survey participants were interviewed by trained field workers on a wide range of reproductive health topics. Response rates were very high at 98% [20]. DHS data are usually weighted to account for sample imbalance and response bias to ultimately ensure that the sample is truly representative of the population.

### Study sample

The analytical samples were young men and women aged 15–24 years. The DHS maintains separate dataset for men, women, couples, children and birth history. Therefore, in consonance with previous studies on reproductive health issues among adolescents, the data for males and females were analysed separately. The inclusion criteria were (1) age 15–24 years; (2) not been declared infecund or sterile; (3) never gave birth to children. After the exclusions, the final weighted sample for men and women were 2674 and 9637 respectively. Previous studies suggested that respondents who have given birth often report fertility desires not less than their number of children ever-born [1, 32]. Therefore, we deliberately excluded young males and females who have given birth to children because childbearing experiences and realities might have altered their fertility desires.

### Analytical framework

The analytical framework for this study was adapted from a theoretical model for fertility behaviour in which the roles of individual and contextual characteristics are clearly delineated [33]. The model was originally founded on the demographic transition theory which stipulated that fertility decline is driven by changes in institutional factors. Some other scholars referred to these as social changes [34, 35]. The adapted framework guided our analytical procedures in this study. Based on past empirical evidence, the guiding hypothesis is that decision on ideal number of children as a measure of fertility desire is shaped by individual, social, economic and ecological or cultural factors [2, 6, 36]. Apart from individual factors, the opportunities and limitations in the environment in which people live affects their reproductive decisions such as ideal number of children. On this basis, the contextual variables analysed in this study includes those that represent the socio-economic environment which comprises urbanization (type of place of residence); geo-political region; community education and occupational profile (extent of unemployment and agricultural work). The list also includes exposure to family planning messages, childhood mortality experience, and attitude to family planning. Unlike many previous studies that focused only on women, we analysed both men and women separately. This is especially important because of the patrilineal nature of Nigeria.

### Description of variables

The dependent variable was “desire for large family size”, alternatively referred to as ideal number of children more than four. During the survey, data on this variable was collected via the question “*if you could choose exactly the number of children to have in your whole life, how many would that be*?” Respondents who desired more than four children were categorized as having desire for large family size (DLFS). The choice of cut-off of four children is based on recommendation of maximum of four children in the Nigeria national population policy [22]. About 5% of the young adults gave non-numeric responses to the question on ideal number of children. These were also categorized as desire for large family size based on previous evidence which suggested that such women invariably prefer large family sizes [37].

Taking cues from the literature, explanatory variables were carefully selected to capture different aspect of “*social contexts*”. For ease of data management, they were grouped into individual, household and community or contextual characteristics. The variables at the individual level included age, highest educational attainment, religion, and ethnicity. The role of education in fertility decisions is well established in the literature. Generally, fertility desire is inversely related to educational attainment [10, 38]. Some studies have shown that fertility desire tends to be higher among certain ethnic groups and religious adherents in Nigeria [21]. Besides, religion and ethnicity are very important indices of “socio-cultural” contexts [39].

At the household level, we included household wealth index and household size. It is anticipated that young men growing up in a “large size” household may desire larger number of children as a reflection of their own household contexts [35]. In the DHS, wealth index was derived using principal component analysis of selected items possessed by households [40]. An inverse relationship is also anticipated between household wealth index and DLFS.

The variables that best represent “social context” were those measured at the community level. In this study, “community” was represented by clusters or enumeration areas which were the primary sampling unit during the implementation of the 2018 NDHS. The first two variables at this level were directly measured and available in the dataset. These were type of place of residence and geo-political region. Place of residence was classified as urban or rural. The literature suggests that fertility desires are usually higher in the rural area [41]. There are six geo-political regions in Nigeria (North Central, North East, North West, South East, South South and South West). The Northern regions especially, North East and North West are known to be massively pronatal and hence the intended number of children is expected to be higher than other regions [21].

Other community-level variables were derived from individual characteristics by aggregating at the cluster level. This is a standard practice in several previous studies where community characteristics have been explored using DHS data [42–44]. The derived community variables and their justification are described as follows:

i. Community education: this was estimated as the proportion of men/women with at least secondary education in the cluster. Literature indicates that community level education is also a significant predictor of fertility [8]. Theoretically, people living in a community with a greater percentage of educated persons are more likely to be aware of the implications of large family sizes and try to avoid such. Also, information about contraceptive choices is more likely in such settings.
ii. Family planning message penetration: this was derived as proportion of participants who have been exposed to family planning messages via radio, television or other media. We expect that this variable would be negatively related to fertility desire [6].
iii. Unemployment: this is the proportion of respondents who were not working (not doing anything to earn personal income) in the community or cluster. Previous studies on labour force participation and fertility suggest a negative relationship [45, 46]. That is, women engaged in formal or informal employment usually have fewer children because of the challenges associated with combining childcare and employment. In a setting, where unemployment is common, women are likely to have or desire large number of children.
iv. Agricultural work: this is the proportion of respondents in the community who were engaged in agriculture-related occupations. One of the main motivations for large family size in the traditional African setting is the notion that children would constitute a dependable workforce for agriculture [47]. This belief may still persist especially in rural areas. It is therefore anticipated that young men and women in such setting are likely to desire large number of children.
v. Child-mortality experience: this variable captured the proportion of men/women in the community who have experienced death of children. This is to capture the age-long “insurance hypothesis” of which one of the argument was that the reason for large families was for fear of child mortality [48]. Some men/women think that if children death occurs, they need to have as many as possible so that they can have people to carry-on their family name. Therefore, we hypothesized that the ideal number of children would be higher in a community or context where childhood mortality is common.
vi. Negative attitude to family planning. Among women, this was calculated as the proportion of women in the community who do not intend to use any method of contraceptive. Overt and covert opposition to family planning is one of the reasons for low contraceptive prevalence in Nigeria and other African countries [49]. For men, those who responded affirmatively to the statements “contraceptive is woman’s business or contraceptive users are promiscuous were categorized as having negative attitude. It can be expected that where this practice is common, people are likely to desire large number of children.

These community (or contextual) variables were derived using the full dataset (for men and women separately) before the inclusion/exclusion criteria described earlier were implemented. In this way, we capture the true contexts in which the young adults who constituted our analytical sample were living at time of data collection.

### Statistical analysis

Sample weight was utilized in all analysis. First, descriptive analysis was done to summarize the study variables. Categorical variables were presented using frequencies and percentages while mean and standard deviation was computed for continuous variables. The second stage involved cross tabulations to explore bivariate relationships between the explanatory variables and DLFS.

At the third stage of analysis, random effect logit models were fitted to explore the relationships further. The choice of this model was informed by the dichotomous distribution of the dependent variable. Also, random effects model permit adequate control for intra cluster correlation occasioned by the hierarchical structure of the DHS data. Two levels of hierarchy were present. These were individual respondents (level 1) nested in communities or enumeration area (level 2). Use of random effects model also helps to ensure that the standard errors of regression coefficients and the associated confidence interval are not biased.

The models were fitted in four steps. The first (model I) was a univariate model in which one explanatory variable was entered at a time. This was followed by model II where individual-level variables were entered into the model. Model III contained all community-level variables. Lastly, model IV contained all explanatory variables with p-values less than 0.05 from models II and III. The main rationale for this sequence was to understand how each group of variables was related to the outcome. The final model also served the purpose of providing a glance of the independent association between community-level variables (social context) and fertility desire having controlled for individual characteristics.

Further analysis was conducted to systematically assess sex differences in factors associated with DLFS. Data for young men and women were combined together. Thereafter, a procedure similar to that followed for men and women was followed to construct the full random-effect logit model that include interaction term for sex (male = 1, female = 0) and each covariate. For all models, measure of effect was reported as Odds Ratio (OR) with their respective 95% Confidence Interval (95% CI). Sex difference was confirmed at instances where the interaction term was statistically significant (p < 0.05). Stata MP Version 14 was used for analyses.

### Ethical approval

In this study, the data analysed is available in the public domain (www.measuredhs.com) after obtaining necessary approvals. Data do not contain any identifying information. The original survey (NDHS 2018) was approved by the National Health Research Ethics Committee in Nigeria (NHREC/01/01/2007).

## Results

### Socio-demographic and contextual characteristics

Characteristics of the young men and women analysed are presented in Table 1. Of the 2674 young men, 35% were aged 20–24 years. Their educational distribution showed that 22% had no formal education while 60% and 9% attained secondary and higher education respectively. More than half (69%) were adherents of Islamic religion. Hausa constituted 44% while Igbo and Yoruba had 10% respectively. Most of the young men were never married (98%). On average, 21% and 17% were from households in the poorest and richest wealth quintile respectively. For community characteristics, 57% lived in rural areas. The largest percentage (38%) was from the North West while North Central, North East, South East, South South and South West had 14%, 21%, 9%, 7% and 11% respectively. Table 1 further show that 36% and 31% of young men reside in places where the community education is rated as low and high respectively. Also, 71% of participants were from communities with low penetration of family planning messages. About one third belonged to communities with high child mortality experience (38%), and negative attitude to family planning (32%).

**Table 1:** Characteristics of non-childbearing young (15–24 years) men and women, Nigeria, 2018

Variables	Male (n = 2674)		Female (n = 9637)	
Individual characteristics	n	%	n	%
Age group				
15–19	1745	65.3	7136	74
20–24	929	34.7	2501	26
Educational attainment				
None	578	21.6	1674	17.4
Primary	263	9.9	834	8.7
Secondary	1601	59.8	6230	64.6
Higher	232	8.7	899	9.3
Religion				
Christianity	825	30.9	4713	48.9
Islam	1834	68.5	4888	50.7
Others	15	0.6	36	0.4
Ethnicity				
Fulani	186	7	494	5.1
Hausa	1189	44.4	2614	27.1
Igbo	276	10.3	1567	16.3
Yoruba	257	9.6	1616	16.8
Others	766	28.7	3346	34.7
Marital status				
Not married	2627	98.2	8358	86.7
Currently married	47	1.8	1279	13.3
Household characteristics				
Household size: mean (sd)	8.9 (4.7)		6.8 (4.3)	
Wealth index				
Poorest	576	21.5	1179	12.2
Poorer	556	20.7	1624	16.9
Middle	536	20.1	1907	19.8
Richer	561	21	2381	24.7
Richest	445	16.7	2546	26.4
Relationship to head of household(women)				
Head	–	–	293	3
Wife	–	–	1132	11.8
Daughter	–	–	6313	65.5
Others	–	–	1898	19.7
Relationship to head of household(men)				
Head	110	4.1	–	–
Son and grandson	2275	85.1	–	–
Brother	104	3.9	–	–
Others	185	6.9	–	–
Community characteristics				
Type of residence				
Urban	1158	43.3	5061	52.5
Rural	1516	56.7	4576	47.5
Region				
North Central	383	14.3	1441	15
North East	573	21.4	1512	15.6
North West	1012	37.8	2571	26.6
South East	234	8.8	1221	12.7
South South	178	6.7	1162	12.1
South West	294	11	1730	18
Community education				
Low	953	35.6	2371	24.6
Medium	907	33.9	3690	38.3
High	814	30.5	3576	37.1
Family planning message penetration				
Low	1887	70.6	6811	70.7
Medium	787	29.4	-	
High	-		2826	29.3
Unemployment				
Low	935	35	2955	30.7
Medium	752	28.1	3415	35.4
High	987	36.9	3267	33.9
Agricultural work				
Low	1010	37.8	3475	36.1
Medium	888	33.2	3621	37.6
High	776	29	2540	26.4
Child mortality experience				
Low	792	29.6	3844	39.9
Medium	878	32.9	2230	33.5
High	1004	37.5	2563	26.6
Negative attitude to family planning				
Low	862	32.2	4094	42.5
Medium	961	35.9	3168	32.9
High	851	31.8	2375	24.7
Ideal number of children				
≤ 4	775	29	4532	47
> 4 (large)	1899	71	5105	53

Among 9637 young women (Table 1, column 3 and 4), majority (74%) were aged 15–19 years. A largest proportion (65.0%) attained secondary education while 9% had higher education. About half were Christians (49%). Distribution of ethnic affiliation showed that Hausa and Yoruba constituted 27% and 17% respectively. Majority (87%) of the young women were unmarried. About one-quarter of the young women were from households in the richest quintile (26%) while only 12% belonged to the poorest quintile. Unlike young men, a slightly larger percentage of young women reside in urban areas (53%). Regional distribution showed that North West (27%) and South West (18%) had the largest share. With respect to other contextual variables, 37% and 34% lived in communities with high education and unemployment respectively. Similarly, 27%, 36% and 25% of young men reside in communities which had high level of child mortality experience, and negative attitude to family planning respectively.

### Fertility desire among young men aged 15–24 years in Nigeria

Overall, 71% of young Nigerian men desired more than four children (large family size). Table 2 Panel 1 shows the distribution of DLFS according to different background variables. There was a wide difference between males with no formal education (89%) and those who had higher education (69%). Similarly, Islamic adherents (85%) tend to had greater proportion with DLFS than Christians (41%). Across ethnic groups, unlike Igbo (38%) and Yoruba (29%), Fulani (91%) and Hausa (87%) had greater proportions with DLFS.

**Table 2:** Desire for large family size (ideal number of children > 4) among young men and women aged 15–24 years in Nigeria, 2018

Variables	Ideal number of children > 4 (Large)			
	Male: (n = 1899)		Female (n = 5105)	
Individual characteristics	n	%	n	%
Age group				
15–19	1227	70.3	3987	55.9
20–24	672	72.4	1118	44.7
Educational attainment				
None	89	89	1424	85.1
Primary	195	74.1	564	67.6
Secondary	1031	64.4	2835	45.5
Higher	159	68.5	283	31.5
Religion				
Christianity	340	41.2	1634	34.7
Islam	1551	84.6	3452	70.6
Others	8	52.2	19	52.3
Ethnicity				
Fulani	169	91	369	74.6
Hausa	1038	87.3	2089	79.9
Igbo	104	37.6	675	43.1
Yoruba	75	29.1	315	19.5
Others	514	67.1	1657	49.5
Marital status				
Not married	1862	70.9	4098	49
Currently married	37	80	1007	78.7
Household characteristics				
Household size: mean(sd)	9.5 (5.0)		7.5 (4.8)	
Wealth index				
Poorest	512	88.9	957	81.2
Poorer	467	84	1151	70.9
Middle	415	77.4	1109	58.1
Richer	321	57.2	1073	45.1
Richest	185	41.5	816	32
Relationship to head of household(women)				
Head	–	–	89	30.5
Wife	–	–	903	79.8
Daughter	–	–	3251	51.5
Others	–	–	862	45.4
Relationship to head of household(men)				
Head	92	84.2	–	–
Son and grandson	1642	72.2	–	–
Brother	63	60.2	–	–
Others	102	54.9	–	–
Community characteristics				
Type of residence				
Urban	711	61.4	2280	45.1
Rural	1188	78.4	2825	61.7
Region				
North Central	274	71.4	651	45.2
North East	509	88.8	1193	78.9
North West	871	86.1	1980	77
South East	96	40.8	586	48
South South	81	45.6	349	23
South West	69	23.6	346	20
Community education				
Low	809	84.9	1952	82.4
Medium	614	67.7	1968	53.3
High	476	58.5	1185	33.1
Family planning message penetration				
Low	1388	73.6	3895	57.2
Medium	511	64.9	–	
High	–		1210	42.8
Unemployment				
Low	763	81.5	1288	43.6
Medium	534	71	1590	46.6
High	603	61.1	2227	68.2
Agricultural work				
Low	612	60.6	1993	57.4
Medium	683	76.9	1748	48.2
High	604	77.8	1364	53.7
Child mortality experience				
Low	406	51.3	1244	32.4
Medium	614	69.9	1837	56.9
High	879	87.6	2024	78.9
Negative attitude to family planning				
Low	518	60.2	1662	40.6
Medium	728	75.7	1704	53.8
High	653	76.8	1739	73.2

Rural dwellers (78%) also had greater DLFS than their urban counterparts (61%). The regional variations show that South West (24%) had the lowest DLFS while North East (89%) and West (81%) ranked topmost. Further, the proportion of young men with DLFS decreased with the levels of community education, family planning message penetration, and unemployment. Conversely, DLFS increased with community-level of agricultural work, child mortality experience and negative attitude to family planning.

### Fertility desire among young women aged 15–24 years in Nigeria

Fifty three percent (53%) of young women in Nigeria desired to have more than four children. Distribution across background socio-economic and contextual variables followed a pattern similar to that described for young men (Table 2, Panel 2). For instance, 20% of young Yoruba women compared to 80% and 75% of Hausa and Fulani desired more than four children. The proportion for rural (62%) was also higher than urban residents (45%). Across regions, 20% of young women in the South West desire more than four children in their life time while the level was 79% and 77% for North East and North West respectively. For South East and South regions, it was 48% and 23%. The pattern of relationship between contextual characteristics and desire for large family size among young women was similar to that of young men. There was a negative relationship with levels of community education, and family planning message penetration while a positive association was observed for community level of unemployment, child mortality experience and negative attitude to family planning.

### Correlates of DLFS among young men in Nigeria

Table 3 shows the unadjusted (Model I) and adjusted (Models II, III and IV) Odds Ratio (OR) for individual and contextual factors associated with DLFS. From Model I, the odds of DLFS was 28% higher among men aged 20–24 years compared to those in age group 15–19 years (OR = 1.28, CI 1.01–1.62). Relative to those with no formal education, young men with higher education (OR = 0.18, CI 0.10–0.31) are less likely to desire large family size. Muslims were 10 times as likely as Christians to desire large family size (OR = 10.1, CI 7.52–13.75). Fulani (OR = 30.48, CI 14.81–62.77) and Hausa (OR = 20.44, CI 12.36–33.81) men relative to Yoruba were much more likely of DLFS. Rural residents were 3 times as likely as their urban counterparts to desire large family size. Those from North East (OR = 5.06, CI 3.14–8.17) and North West (OR = 3.33, CI 2.18–5.11) regions were more likely of DLFS compared to those from North Central region. In contrast, respondents from South East, South South and South West regions were less likely of DLFS. Young men from communities with high education were less likely than those in communities with low education. Other community-level characteristics positively associated with DLFS include high level of agricultural work (OR = 4.26, CI 2.68–6.76), child mortality experience (OR = 12.64, CI 7.90–20.22) and negative attitude to family planning (OR = 3.09, CI 1.96–4.89).

**Table 3:** Factors associated with desire for large family size among young men aged 15–24 years in Nigeria, 2018

Variables	Model I	Model II	Model III	Model IV
Individual characteristics	OR_unadj_ (95% CI)	OR_adj_ (95% CI)	OR_adj_ (95% CI)	OR_adj_ (95% CI)
Age group				
15–19	1	1		
20–24	1.28 (1.01–1.62)*	1.14 (0.89–1.46)		
Educational attainment				
None	1	1		
Primary	0.24 (0.14–0.41)*	0.71 (0.42–1.19)		
Secondary	0.16 (0.09–0.24)*	0.79 (0.51–1.23)		
Higher	0.18 (0.10–0.31)*	0.90 (0.50–1.62)		
Religion				
Christianity	1	1		1
Islam	10.1 (7.52–13.75)*	4.57 (3.15–6.62)*		3.95 (2.68–5.83)*
Others	1.38 (0.29–6.58)	0.85 (0.19–3.89)		0.67 (0.15–2.91)
Ethnicity				
Fulani	30.48 (14.81–62.77)*	6.60 (3.26–13.39)		2.09 (0.93–4.71)
Hausa	20.44 (12.36–33.81)*	5.11 (3.13–8.35)*		1.79 (0.92–3.50)
Igbo	1.25 (0.72–2.17)	2.87 (1.65–4.97)*		1.43 (0.61–3.38)
Yoruba	1	1		1
Others	5.85 (3.63–9.42)*	4.52 (2.85–7.21)*		1.78 (0.98–3.24)
Marital status				
Not married	1	1		
Currently married	1.57 (0.56–4.38)	0.58 (0.19–1.76)		
Household characteristics				
Household size:	1.10 (1.07–1.13)*	1.06 (1.03–1.09)*		1.05 (1.02–1.09)*
Wealth index				
Poorest	1	1		1
Poorer	0.49 (0.32–0.78)*	0.82 (0.53–1.27)		0.92 (0.60–1.40)
Middle	0.29 (0.18–0.45)*	0.70 (0.45–1.09)		0.79 (0.51–1.23)
Richer	0.13 (0.08–0.21)*	0.37 (0.23–0.59)*		0.47 (0.29–0.75)*
Richest	0.06 (0.03–0.09)*	0.18 (0.11–0.30)*		0.28 (0.16–0.75)*
Relationship to head of household(men)				
Head	1	1		1
Son and grandson	0.42 (0.22–0.81)*	0.35 (0.17–0.69)*		0.38 (0.20–0.72)*
Brother	0.35 (0.15–0.80)*	0.40 (0.17–0.94)*		0.41 (0.19–0.92)*
Others	0.33 (0.16–0.71)*	0.46 (0.21–1.01)		0.55 (0.27–1.13)
Community characteristics				
Type of residence				
Urban	1		1	
Rural	3.17 (2.15–4.67)*		1.01 (0.73–1.40)	
Region				
North Central	1		1	1
North East	5.06 (3.14–8.17)*		2.79 (1.66–4.68)*	1.38 (0.84–2.25)
North West	3.33 (2.18–5.11)*		2.19 (1.37–4.99)*	1.23 (0.73–2.08)
South East	0.24 (0.15–0.40)*		0.29 (0.17–0.48)*	0.93 (0.42–2.06)
South South	0.38 (0.22–0.65)*		0.44 (0.25–0.77)*	1.18 (0.69–2.00)
South West	0.10 (0.06–0.17)*		0.11 (0.06–0.18)*	0.28 (0.16–0.50)*
Community education				
Low	1		1	
Medium	0.22 (0.14–0.35)*		0.82 (0.56–1.21)	
High	0.12 (0.07–0.19)*		0.85 (0.56–1.29)	
Family planning message penetration				
Low	1		1	
Medium	0.73 (0.56–0.96)*		0.90 (0.69–1.16)	
High	–		–	
Unemployment				
Low	1		1	
Medium	0.41 (0.26–0.65)*		1.01 (0.69–1.47)	
High	0.21 (0.14–0.33)*		1.15 (0.77–1.74)	
Agricultural work				
Low	1		1	1
Medium	2.47 (1.57–3.89)*		1.33 (0.92–1.92)	1.12 (0.79–1.57)
High	4.26 (2.68–6.76)*		1.72 (1.10–2.67)*	1.18 (0.79–1.75)
Child mortality experience				
Low	1		1	1
Medium	2.62 (1.73–3.94)*		1.40 (1.02–1.97)*	1.26 (0.93–1.70)
High	12.64 (7.90–20.22)*		1.75 (1.11–2.74)*	1.22 (0.81–1.83)
Negative attitude to family planning				
Low	1		1	1
Medium	2.80 (1.79–4.39)*		1.79 (1.27–2.53)*	1.77 (1.30–2.42)*
High	3.09 (1.96–4.89)*		1.64 (1.13–2.38)*	1.72 (1.23–2.40)*

* *p* < 0.05

Model II (Table 3) shows the association of individual-level variables with desire for large family size. Islam religion, ethnicity, household size and wealth quintile retained their positive associations. Young Muslim males were 5 times as likely as Christians (OR = 5.11, CI 3.13–8.35) to desire large families. Similarly, Fulanis and Hausas were more likely than Yorubas to desire more than four children. A unit increase in household size was associated with 6% higher odds of DLFS. Participants with households in the richer (OR = 0.37, CI 0.23–0.59) and richest quintile (OR = 0.18, CI 0.11–0.30) were less likely than those in the poorest quintile to desire large family.

In Model III (Table 3), the contextual factors significantly related to large family size desire include geopolitical region, community levels of agricultural work, child mortality experience, and negative attitude to family planning. The direction of influence of these variables remained as observed in Model I but with lesser magnitude.

Model IV shows that the significant correlates of DLFS were religion, household size, wealth quintile, relationship to head of household, geopolitical region, community levels of negative attitude to family planning. Specifically, young Muslim men were 4 times as likely as their Christian counterparts to desire large family size (OR = 3.95, CI 2.68–5.83). For geo-political region, significant differentials in desire for large family was observed for only the Southwest region (OR = 0.28, CI 0.16–0.50) relative to the North Central. Lastly, in communities with medium (OR = 1.77, CI 1.30–2.42) or high (OR = 1.72, CI 1.23–2.40) negative attitude to family planning, young men were more likely of DLFS compared to where such attitude is low.

### Correlates of DLFS among young women in Nigeria

Results for women (Table 4) showed that the unadjusted association between many of individual as well as contextual factors and desire for large family size among young women was similar in direction to those of young men reported previously. In model I, young women aged 20–24 years were less likely of DLFS compared to those aged 15–19 years (OR = 0.88, CI: 0.78–0.99). Young Muslim women (OR = 5.02, CI 4.35–5.83) were 5 times as likely as Christians to desire large family sizes. Furthermore, Fulani (OR = 16.69, CI 12.05–23.11) and Hausa (OR = 22.48, CI 17.68–28.95) women were significantly more likely of DLFS compared to Yorubas. Household size was positively associated with the likelihood of DLFS while a negative trend was observed for household wealth quintile. The odds of DLFS was higher among women from North East (OR = 6.19, CI 4.81–7.95) and North West (OR = 7.18, CI 5.64–9.14) relative to South West region while it was lesser in South South (OR = 0.54, CI 0.42–0.69). The contextual characteristics retained the pattern of relationships as reported for young men. Strong influence was observed for high community unemployment (OR = 3.79, CI 2.97–4.85), child mortality experience (OR = 11.41, CI 9.08–14.35) and negative attitude to family planning (OR = 5.94, CI 4.68–7.54).

**Table 4:** Factors associated with desire for large family size among young women aged 15-24 years in Nigeria, 2018

Variables	Model I	Model II	Model III	Model IV
Individual characteristics	OR_adj_ (95% CI)	OR_adj_ (95% CI)	OR_adj_ (95% CI)	OR_adj_ (95% CI)
Age group				
15–19	1	1		
20–24	0.88 (0.78–0.99)*	1.09 (0.96–1.24)		
Educational attainment				
None	1	1		1
Primary	0.38 (0.29–0.48)*	0.84 (0.66–1.07)		0.99 (0.77–1.27)
Secondary	0.18 (0.15–0.22)*	0.63 (0.51–0.78)*		0.78 (0.62–0.97)*
Higher	0.09 (0.08–0.13)*	0.43 (0.33–0.58)*		0.53 (0.39–0.70)*
Religion				
Christianity	1	1		1
Islam	5.02 (4.33–5.83)*	2.83 (2.39–3.34)*		2.19 (1.82–2.64)*
Others	0.77 (0.36–1.64)	0.70 (0.35–1.39)		0.67 (0.33–1.36)
Ethnicity				
Fulani	16.69 (12.05–23.11)*	5.03 (3.65–6.92)*		2.48 (1.70–3.61)*
Hausa	22.48 (17.46–28.95)*	6.37 (4.98–8.15)*		2.95 (2.11–4.13)*
Igbo	3.63 (2.82–4.68)*	5.47 (4.29–6.95)*		2.19 (1.52–3.17)*
Yoruba	1	1		1
Others	4.85 (3.88–6.06)*	3.59 (2.91–4.44)*		2.09 (1.57–2.78)*
Marital status				
Not married	1	1		1
Currently married	2.93 (2.43–3.53)*	1.50 (1.03–2.19)*		1.47 (1.00–2.15)*
Household characteristics				
Household size:	1.04 (1.02–1.05)*	1.04 (1.03–1.06)*		1.03 (1.01–1.05)*
Wealth index				
Poorest	1	1		1
Poorer	0.56 (0.45–0.69)*	0.87 (0.70–1.07)		1.02 (0.83–1.27)
Middle	0.30 (0.24–0.38)*	0.62 (0.49–0.76)*		0.83 (0.67–1.04)
Richer	0.21 (0.16–0.26)*	0.47 (0.37–0.58)*		0.71 (0.56–0.90)*
Richest	0.12 (0.09–0.15)*	0.31 (0.24–0.39)*		0.54 (0.41–0.69)*
Relationship to head of household(women)				
Head	1	1		
Wife	4.69 (3.23–6.81)*	1.38 (0.85–2.22)		
Daughter	1.59 (1.14–2.19)*	1.06 (0.75–1.48)		
Others	1.62 (1.16–2.27)*	1.21 (0.86–1.71)		
Community characteristics				
Type of residence				
Urban	1		1	
Rural	2.74 (2.24–3.35)*		0.95 (0.81–1.11)	
Region				
North Central	1		1	1
North East	6.19 (4.81–7.95)*		4.03 (3.19–5.10)*	2.78 (2.20–3.48)*
North West	7.18 (5.64–9.14)*		4.74 (3.66–6.16)*	2.61 (1.98–3.45)*
South East	1.25 (0.98–1.59)		2.01 (1.58–2.57)*	2.30 (1.61–3.29)*
South South	0.54 (0.42–0.69)*		0.81 (0.63–1.04)	1.06 (0.83–1.35)
South West	0.25 (0.19–0.32)*		0.44 (0.34–0.56)*	0.81 (0.59–1.09)
Community education				
Low	1		1	1
Medium	0.14 (0.11–0.18)*		0.53 (0.43–0.66)*	0.80 (0.64–1.01)
High	0.07 (0.05–0.08)*		0.42 (0.33–0.55)*	0.68 (0.52–0.89)*
Family planning message penetration				
Low	1		1	1
Medium	–			
High	0.77 (0.68–0.87)*		0.87 (0.78–0.98)*	0.94 (0.83–1.06)
Unemployment				
Low	1		1	
Medium	1.11 (0.88–1.39)		0.88 (0.75–1.04)	
High	3.79 (2.97–4.85)*		1.09 (0.89–1.33)	
Agricultural work				
Low	1		1	1
Medium	0.53 (0.41–0.68)*		1.06 (0.89–1.27)	1.10 (0.93–1.31)
High	0.71 (0.56–0.92)*		1.39 (1.14–1.72)*	1.40 (1.15–1.71)*
Child mortality experience				
Low	1		1	1
Medium	2.78 (2.28–3.41)*		1.56 (1.33–1.83)*	1.29 (1.11–1.51)*
High	11.41 (9.08–14.35)*		1.44 (1.14–1.82)*	1.08 (0.86–1.34)
Negative attitude to family planning				
Low	1		1	1
Medium	2.29 (1.84–2.87)*		1.62 (1.38–1.90)*	1.41 (1.21–1.64)*
High	5.94 (4.68–7.54)*		1.88 (1.55–2.28)*	1.36 (1.13–1.65)*

* *p* < 0.05

Model II for individual characteristics showed that education, religion, ethnicity, marital status, household size and wealth index remained as significant correlates of DLFS. However, the strength of association was reduced for most of the variables. For instance, Islamic women were still more likely of DFLS than Christians (OR = 2.83, CI 2.39–3.34); ditto for Fulani (OR = 5.03, CI 3.65–6.92) and Hausa (OR = 6.37, CI 4.98–8.15) compared to Yoruba women. Women from households in the richer and richest quintile were still less likely than those from the poorest quintile to desire large family size.

Model III for contextual variables showed that geo-political region, community education, family planning message penetration, agricultural work, child mortality experience, and negative attitude to family planning retained a significant relationship with DLFS (Table 4).

In the final model, individual-level factors related to large family size desire included educational attainment, religion, ethnicity, household size and wealth quintile. Young women who had attained secondary (OR = 0.78, CI 0.62–0.97) and higher education (OR = 0.53, CI 0.39–0.70) were less likely than those with no formal education to desire large family size. Muslim women were twice as likely as their Christian counterparts to desire large family size. Further, the odds of DLFS decreased with wealth quintile while it increased with household size (OR = 1.03, CI 1.01–1.05). The significant contextual variables were region, community education, agricultural work, child mortality experience and negative attitude to family planning. Young women from the North East (OR = 2.78, CI 2.20–3.48), North West (OR = 2.61, CI 1.61–3.29) and South East (OR = 2.30, CI 1.61–3.29) were more likely of DLFS than those in North Central region. South South and South West regions were not significantly different from the North central. In addition, women who lived in communities with high level of education were less likely to desire large family size unlike those from low community education settings. Where agricultural work was highly prevalent, there was greater likelihood for young women to desire large family size (OR = 1.41, CI 1.15–1.71). Same pattern was observed for medium child mortality experience (OR = 1.29, CI 1.11–1.51) and high negative attitude to family planning (OR = 1.36, CI 1.13–1.65).

### Sex differences in the correlates of DLFS among young men and women

Table 5 presents results for correlates of desire for large family size among young men and women in Nigeria. Most of the variables retained their direction of relationship as reported from the separate models for men and women. Further tests of sex differences in the correlates are summarized in Table 5 column 3. Three factors showed differential influences between men and women. These include religion, richest wealth quintile, and community level of negative attitude to family planning. The effect size for Islam versus Christianity among young men was almost twice those of young women (OR = 2.74, CI 1.85–4.05). Conversely, the size of effect of richest versus poorest wealth quintile on DLFS among young men was less than those of young women (OR = 0.41, CI 0.24–0.72). In addition, medium (OR = 1.75, CI 1.28–2.40) and high (OR = 1.51, CI 1.07–2.12) negative attitude to family planning had bigger effect sizes among men than women.

**Table 5:** Assessment of sex difference in factors associated with desire for large family size among young men and women in Nigeria, 2018

Variables	Model for men and women	Sex differences+ +
Individual characteristics	OR_adj_ (95% CI)	OR_adj_ (95% CI)
Sex		
Male	1.74 (1.51–2.02)*	**–**
Female	1	**–**
Age group		
15–19	1	1
20–24	1.15 (1.03–1.28)*	1.20 (0.94–1.54)
Educational attainment		
None	1	1
Primary	0.93 (0.74–1.16)	0.76 (0.44–1.32)
Secondary	0.76 (0.63–0.93)*	0.87 (0.53–1.41)
Higher	0.55 (0.43–0.72)*	1.04 (0.56–1.93)
Religion		
Christianity	1	1
Islam	2.47 (2.09–2.92)*	2.74 (1.85–4.05)*
Others	0.63 (0.34–1.18)	0.41 (0.09–1.88)
Ethnicity		
Fulani	2.35 (1.68–3.29)*	1.24 (0.55–2.82)
Hausa	2.61 (1.94–3.51)*	1.03 (0.53–2.03)
Igbo	2.00 (1.43–2.79)*	1.11 (0.46–2.69)
Yoruba	1	1
Others	1.98 (1.53–2.55)*	1.16 (0.64–2.12)
Marital status		
Not married	1	1
Currently married	1.61 (1.33–1.95)*	0.81 (0.30–2.18)
Household characteristics		
Household size:	1.03 (1.01–1.04)*	
Wealth index		
Poorest	1	1
Poorer	0.98 (0.81–1.18)	0.99 (0.63–1.55)
Middle	0.79 (0.65–0.96)*	0.82 (0.51–1.33)
Richer	0.63 (0.51–0.77)*	0.60 (0.36–0.99)
Richest	0.47 (0.38–0.59)*	0.41 (0.24–0.72)*
Community characteristics		
Type of residence		
Urban		
Rural		
Region		
North Central	1	1
North East	2.62 (0.12–3.22)*	0.76 (0.46–1.25)
North West	2.39 (1.87–3.06)*	0.78 (0.46–1.31)
South East	1.92 (1.39–2.65)*	0.49 (0.21–1.10)
South South	1.03 (0.83–1.28)	1.51 (0.87–2.61)
South West	0.63 (0.48–0.82)*	0.32 (0.19–0.58)
Community education		
Low	1	1
Medium	0.98 (0.81–1.18)	1.27 (0.85–1.89)
High	0.94 (0.83–1.05)	1.51 (0.97–2.33)
Family planning message penetration		
Low	1	1
Medium	0.86 (0.69–1.08)	0.94 (0.72–1.22)
High	0.94 (0.83–1.05)	
Agricultural work		
Low	1	1
Medium	1.17 (1.02–1.34)*	1.27 (0.89–1.78)
High	1.39 (1.18–1.63)*	1.38 (0.92–2.06)
Child mortality experience		
Low	1	1
Medium	1.23 (1.08–1.39)*	1.14 (0.84–1.54)
High	1.03 (0.86–1.23)	1.04 (0.69–1.56)
Negative attitude to family planning		
Low	1	1
Medium	1.42 (1.25–1.61)*	1.75 (1.28–2.40)*
High	1.43 (1.23–1.65)*	1.51 (1.07–2.12)*

+ + from interaction model

* *p* < 0.05

## Discussion

This study aimed at exploring the social contexts (individual, household and contextual characteristics) associated with fertility desires among non-childbearing young adults in Nigeria. Our results revealed that 71% and 53% of young Nigerian men and women respectively desired more than four children. Factors positively associated with desire for large family sizes among young males and females include Islam religion, household size, and wealth index. Community-level factors such as negative attitude to family planning and child mortality experience were positively associated with DLFS among females. Our results showing strong preference for large number of children among young men and women are in congruent with evidence in the literature [20]. This implies that the norm of large family size may not wane even among the younger generation notwithstanding their peculiar opportunities and challenges occasioned by harsh economic environment. However, it is useful to observe that until recent times, apart from mere policy recommendation on ideal number of children, not much has been done to address desire for large family sizes. Reproductive health programmes have prioritized maternal and child healthcare uptake and use of family planning to reduce unwanted pregnancies and its associated consequences. Although some of the programmes may indirectly impact on fertility desire, the persistence of preference for large families is a wakeup call for targeted interventions. Most importantly, a critical question that needs urgent answers is “why do young people in Nigeria still want large families?” Without any active communication and programmatic intervention, large family size preference may persist for a longer time. This would slow down the much-desired transition to medium–low fertility. The multiplier effects on population development and socio-economic and health indices have a worrisome outlook.

At the individual level, among young women, the likelihood of large family size desire decreased with educational attainment. This reflects the known pattern of relationship between education and fertility levels [50, 51]. The result is also in conformity with patterns observed about factors associated with desired family size in other developing countries [2]. Young women with secondary or higher education would be looking forward to formal employment; knowing fully well that it may be herculean to combine their job with childcare, they might have consciously opted for fewer numbers of children. In addition, having experienced the requirements and sacrifices to get formal education, this firsthand exposure may have dissuaded them from the norm of large family size. Among young men, educational attainment was not a significant factor, though secondary and higher education was associated with lesser propensity for large family size.

Other significant factors among men and women included religion. The odds of large family size desire were lesser among Christians than Muslims. These also concur with results from previous fertility studies [21, 28, 29]. The differences observed between Christians and Muslims could be due to variations in their dispositions to polygamy. Islam and traditional religion favour polygamy which is a major motivation for larger number of children.

Ethnicity was statistically significant among women in which case Hausas and Fulani women have a stronger tendency to desire large families. This is likely to reflect variations in the status or position of women across the various ethnic groups [26, 52]. In Nigeria, all ethnic groups and religions believe in procreation. Ethnic differentials in DLFS between Yorubas and others may be an aftermath of early exposure to western education and civilization in the former. This supposition is well supported by the diffusion theory [53].

At the household level, both size and wealth quintile were significant determinants of desire for large family size among unmarried young adults in Nigeria. As household wealth quintile increased, DFLS decreased. For household size, it exhibited a positive association with the outcome variable. Negative relationship between socio-economic indices and family size aspirations has been reported in previous studies. It remains a conundrum why persons in poor/poorer households would desire larger family sizes. The case of household size is understandable. Young adults may form their own expectations based on the household environment in which they are growing up. In other words, they may simply want to emulate their parents in terms of family formation and preferences. Family norms and practices have strong influence in different parts of Nigeria [26, 54–56].

For both of men and women, regional differences between the South West and other regions were obvious. This has been the persistent patterns in several demographic and reproductive health indices in Nigeria [28, 29, 42, 57, 58]. Consequently, there have been repeated recommendations that intervention programmes be tailored to the peculiarities of each geo-political region. Indeed, there are lots of intervention efforts ongoing in North East and North West regions in response to the specific challenges in these regions. Among women, community education was negatively related to DLFS. This is quite intuitive because it is expected that where education is high, fertility desires would be low [17].

Among women, child mortality experience is well known to be associated with propensity to have many children. This is the “insurance phenomenon” of the fertility replacement hypothesis [59] which posits that women may want to have many children so that in case any of them die, the survivors would still be close to their desired family size. To achieve a drastic downward review in ideal number of children which ultimately can lead to lower fertility level; childhood mortality must be sufficiently reduced.

Negative attitude to family planning at the community level was a significant correlate of DLFS. Results for this variable should be interpreted with caution because this study utilized cross-sectional data. As such, only association and not causality can be inferred from the models. The result for attitude to family planning simply illustrated its association with fertility aspirations. There would be opposition to family planning where people want many children. To break this opposition barrier, there should be strong motivation for smaller families.

Sex-specific differences between men and women were observed in the effect of Islamic religion, richest household wealth quintile and negative attitude to family planning. For the three variables, the effect sizes were larger among men than women. For example, this implies that Islam exerts stronger influence on DLFS among men than women. These results reflect the dominant roles of men in reproductive decision making which is not a surprise in the Nigeria setting that is deeply patriarchal [26, 56].

These sex differences may have also been driven by gender dynamics. Previous studies have shown relationship between gender role attitudes, women empowerment and reproductive health behaviour [60, 61]. These studies demonstrated that women with better empowerment or autonomy tend to exhibit better reproductive behaviour [62, 63]. However, these studies were conducted among married women in broad reproductive age group 15–49 because the data on empowerment and gender roles are usually collected as part of the domestic violence module which is strictly for married women. Unfortunately, the issues surrounding gender norms could not be explored in this study given our focus on young men and women who were mostly unmarried.

One limitation in this study is the inability to explore the motivations for desired family size. Though, some factors associated with fertility desire have been identified; however, understanding the mechanism underlying the relationships is well beyond the scope of the data. We could not include other indices of social contexts such as socio-cultural norms and values that inform fertility decisions. Data on these variables were not part of the variables collected for demographic and health surveys. Also, we could not control for prior exposure to reproductive health education which has been implemented in many States of Nigeria. On a positive note, this study has set a benchmark for future studies on fertility desires or aspirations among young people. It improved on previous fertility studies in three ways. First, both males and females were included unlike many others that focused only on females. Second, we objectively investigated sex differences in determinants of fertility desires among young people. Third, beyond individual-level factors, household and contextual factors were explored as determinants of fertility desire. Some factors amenable to programmatic interventions on family size aspirations among young persons have been brought to light.

## Conclusions

Young men and women in Nigeria desire large family sizes but it is much more prevalent among men. Apparently, many young men and women desired to exceed four children recommended in the country’s population policy. Religious affiliation is a strong individual-level correlate of desire for large family size. Contextual characteristics such as community education, attitude to family planning and childhood mortality experience are also related to family size desires among women.

The level of desired family size among young adults is worrisome for fertility transition in Nigeria. The consequence is explosive population growth without commensurate expansion in social infrastructure and other opportunities for young people who are economically active. Subsequently, the popular concept of “demographic dividend” may become “demographic liability”.

Massive improvement in educational enrollment for females is a key strategy to accelerate the fertility transition in Nigeria. This has virtually become a demographic and developmental standard based on experience from several developing countries. It may be worthwhile to include benefits of smaller families in civic education package for young people. This is necessary because it is obvious that there is a need for communication intervention on desired family size among young adults in Nigeria. Though, religion and ethnicity are individual-level characteristics, they duly represent ‘social contexts’. Although these variables are not modifiable, however, religious bodies and ethno-cultural organizations have key roles to play in advocacy and sensitization on benefits of smaller family sizes. The next revision of the Nigeria population policy should be deliberately strategic in providing directions on ideal family size and inclusion of religious and socio-cultural stakeholders.

The importance of a number of community-level factors demonstrated in this paper which is in tandem with previous studies [8] further showed that community-level advocacy and interventions will be necessary to dissuade young people from pronatalism. Further, sex differences in some factors associated with DLFS implied that specific strategies may be needed for males and females. For instance, faith-based and ethno-cultural organisations may be strategic partners for community outreaches targeted at men. At programmatic levels, there is need for sensitization, advocacy and campaigns on family planning to be expanded and sustained. This is because the negative attitude to family planning has to be neutralized. Interventional studies along this line have shown some promising results that can be translated into programme actions [64, 65]. Child survival programmes and interventions should continue to be promoted. Once women see that children are surviving better, they may review their desired family size downward. In summary, a social context favourable to desire for fewer number of children should be created in Nigeria to achieve a faster transition to lower fertility. One question of importance for further research is how do young men and women form fertility expectations about number of children? In addition, the gender dimensions of motivations for large family sizes also deserve further inquiries. Answers to this question can inform relevant interventions at various levels.

## Data Availability

The datasets analysed for the current study are available in the Measure DHS program repository [66].

## Abbreviations

CI: Confidence Interval
DFS: Desired Family Size
DLFS: Desire For Large Family Sizes
DHS: Demographic and Health Survey
NDHS: Nigeria Demographic and Health Survey
NHREC: National Health Research Ethics Committee
OR: Odds Ratio
